# PART1 facilitates tumorigenesis and inhibits ferroptosis by regulating the miR-490-3p/SLC7A11 axis in hepatocellular carcinoma

**DOI:** 10.18632/aging.206009

**Published:** 2024-07-05

**Authors:** Decheng Li, Meiling Wan, Xiaoling Liu, Suvash Chandra Ojha, Yunjian Sheng, Yaling Li, Changfeng Sun, Cunliang Deng

**Affiliations:** 1 Department of Infectious Diseases, The Affiliated Hospital, Southwest Medical University, Luzhou, Sichuan 646000, China; 2 Laboratory of Infection and Immunity, The Affiliated Hospital, Southwest Medical University, Luzhou, Sichuan 646000, China; 3 Department of Pharmacy, The Affiliated Hospital, Southwest Medical University, Luzhou, Sichuan 646000, China

**Keywords:** hepatocellular carcinoma, ferroptosis, PART1, miR-490-3p, SLC7A11

## Abstract

Background: Ferroptosis is associated with cancer progression and has a promising application for treating hepatocellular carcinoma (HCC). Long non-coding RNA (lncRNA) participates widely in the regulation of ferroptosis, but the key lncRNA regulators implicated in ferroptosis and their molecular mechanisms remain to be identified.

Methods: Bioinformatic analysis was performed in R based on The Cancer Genome Atlas Program (TCGA) public database. The relative expression of genes was detected by real-time quantitative PCR. Cell viability was assessed by the CCK8 assay. The cell cycle and apoptosis were detected by flow cytometry. Migration and invasion of HCC cells were detected by Transwell assay and wound healing assay. Expression of relevant proteins was detected by Western blotting. A dual-luciferase reporter assay was used to detect interactions between PART1 (or SLC7A11) and miR-490-3p.

Results: The PART1/miR-490-3p/SLC7A11 axis was identified as a potential regulatory pathway of ferroptosis in HCC. PART1 silencing reduced HCC cell proliferation, migration, and metastasis and promoted apoptosis and erastin-reduced ferroptosis. Further investigation revealed that PART1 acted as a competitive endogenous RNA (ceRNA) for miR-490-3p to enhance SLC7A11 expression. Overexpression of miR-490-3p downregulated the expression of SLC7A11, inhibiting the proliferation, invasion, and metastasis of HCC cells while promoting apoptosis and erastin-induced ferroptosis. Knockdown of PART1 in HCC cells significantly improved the sensitivity of HCC cells to sorafenib.

Conclusion: Our results revealed that the PART1/miR-490-3p/SLC7A11 axis enhances HCC cell malignancy and suppresses ferroptosis, which provides a new perspective for understanding of the function of long chain non-coding RNAs in HCC. The PART1/miR-490-3p/SLC7A11 axis may be target for improving sorafenib sensitivity in HCC.

## INTRODUCTION

In 2020, hepatocellular carcinoma (HCC) ranked as the sixth most diagnosed cancer and the third leading cause of cancer-related death worldwide [1]. Compared to other cancers, HCC has a poorer prognosis, with a 5-year survival rate of only 12.1%, and the median survival time for patients with advanced liver cancer is only approximately 1 year. Ferroptosis is a complex process of programed cell death that is distinct from apoptosis, necroptosis, pyroptosis, and autophagy [2–4], which is involved in the pathogenesis of cancer and has a promising application in the treatment of HCC [5–7]. A thorough investigation of the mechanisms of ferroptosis in HCC may reveal novel molecular targets for therapy. However, the complete elucidation of the molecular mechanism governing ferroptosis and drug resistance in HCC remains elusive.

Long non-coding RNAs (lncRNAs) are defined as RNA transcripts that are longer than 200 nucleotides in length, have no protein-coding potential [8–11], and play essential roles in various biological processes and diseases [12]. LncRNAs share binding sites with miRNAs and compete to act as competing endogenous RNAs (ceRNAs) to further regulate target genes in cancer progression [13–15].

The lncRNA prostate androgen-regulated transcript 1 (PART1) has recently been implicated in the pathogenesis of various cancers [16]. In HCC, PART1 has been demonstrated to promote HCC progression by targeting the miR-590-3p/HMGB2 axis [17], and to promote proliferation, migration, and invasion by targeting the miR-149-5p/MAP2K1 axis [18]; it has been shown to enhance the proliferation and differentiation of Hep3B cells by targeting the miR-3529-3p/FOXC2 axis [16]. In the present study, we constructed a ferroptosis-associated ceRNA network by data mining and found that PART1 may be involved in the regulation of ferroptosis in HCC through the miR-490-3p/SLC7A11 axis. However, the effect of PART1 as a ceRNA on ferroptosis during the progression of HCC has not yet been elucidated.

In the axis, miR-490-3p is considered a critical microRNA that suppresses HCC cell proliferation and migration by targeting AURKA [19], TOMOD3 [20], PPM1F [21], or TNKS2 [22]. Solute carrier family 7 membrane 11 (SLC7A11), a 12-pass transmembrane protein, is a component of the amino acid transporter system xc^–^ and a critical negative regulator of ferroptosis [23, 24]. Downregulation or deficiency of SLC7A11 leads to intracellular cysteine depletion, hindering glutathione (GSH) biosynthesis, which indirectly leads to inhibition of glutathione peroxidase 4 (GPX4) activity and results in lipid peroxide accumulation, triggering ferroptosis. Conversely, high expression of SLC7A11 protects cancer cells from oxidative stress and iron toxicity by promoting cystine uptake and synthesis of reduced GSH. SLC7A11 has been shown to promote drug resistance, chemo-, and radioresistance in various cancers. For example, overexpression of SLC7A11 decreases the sensitivity of gliomas to temozolomide [25, 26] and renders melanoma resistant to BRAF inhibitors [27], whereas downregulation of SLC7A11 expression or blockade of SLC7A11 activity with specific inhibitors such as sulfasalazine (SSZ) effectively enhances the cytotoxicity of cisplatin or doxorubicin in colorectal, bladder, and triple-negative breast cancer cells [28–31]. Lung cancers with mutations in KEAP1 (an important tumor suppressor gene) are known to be radiotherapy-resistant, which highlights the key role of SLC7A11 in radioresistance. Mutations or defects in KEAP1 in lung cancer cells result in constitutive activation of NRF2 and aberrant expression of NRF2 transcriptional targets, including SLC7A11 [32]. NRF2 is a key transcription factor in the antioxidant response to ubiquitination and proteasomal degradation. SLC7A11 expression induced by KEAP1 deficiency or NRF2 overexpression have been shown to attenuate high-energy ionizing radiation (IR)-induced lipid peroxidation and promote radioresistance by inhibiting ferroptosis, demonstrating the potential of the NRF2/SLC7A11/ferroptosis axis as a target against therapeutic resistance [32, 33]. In HCC, Chen et al. found that SOCS2 acts as a bridge to transfer attached ubiquitin to SLC7A11 and promotes K48-linked polyubiquitination degradation of SLC7A11, ultimately leading to ferroptosis and radiosensitization [34]. Evidence suggests that SLC7A11 overexpression or upregulation inhibits ferroptosis (including sorafenib-induced ferroptosis) and renders HCC cells resistant to sorafenib [35]. However, the regulatory mechanism of SLC7A11 remains unclear. Therefore, in this study, we aimed to elucidate whether PART1 is involved in the regulation of ferroptosis in HCC cells by modulating the miR-490-3p/SLC7A11 axis and affecting the sensitivity to sorafenib, which may provide an important theoretical basis for understanding the mechanism of HCC progression and ferroptosis, as well as providing a potential therapeutic target for the treatment of HCC.

## MATERIALS AND METHODS

### Bioinformatics analysis

The ferroptosis-associated gene set obtained from the gene set enrichment analysis (GSEA) database (https://www.gsea-msigdb.org/gsea/index.jsp), contains 64 genes. To identify the differentially expressed genes (DEGs) between HCC tissues and normal tissues, RNA sequencing data were downloaded from The Cancer Genome Atlas Program (TCGA) data portal (https://tcga-data.nci.nih.gov/tcga/), including 374 liver cancer tissues and 50 normal liver tissues. DEGs were identified using the “limma” package in R. The screening criteria for DEGs were set as |log2Foldchange (FC)| >1 and *P*-value < 0.05. To analyze the correlation between ferroptosis-related genes and prognosis of HCC patients, the clinical information of HCC patients with survival time >30 days was downloaded and used to perform survival analysis by the “survival” package in R. To further explore the role of lncRNA-based ceRNA network in regulating ferroptosis in HCC, the miRNA sequencing data of HCC patients were also downloaded from the TCGA database to screen for differentially expressed miRNAs. Then, the interaction between mRNA-mRNA, miRNA-mRNA, and lncRNA-miRNA was established in the STRING (https://cn.string-db.org/), TargetScanHuman (https://www.targetscan.org/vert_72/), DIANA (https://diana.e-ce.uth.gr/lncbasev2/ interactions) tools, respectively, and finally visualized in Cytoscape 3.8 software.

### Cell culture and transient transfection

The normal hepatocyte line LO2 and the human HCC cell lines HCCLM3 and Huh7 were purchased from Procell Life Science and Technology Co., Ltd. (Wuhan, China).

Cells were cultured in Dulbecco’s modified Eagle medium (DMEM) supplemented with 10% fetal bovine serum (FBS) in a humidified incubator at 37°C with 5% CO2, and were periodically checked for mycoplasma contamination. If necessary, penicillin-streptomycin solution (100×) was added to prevent bacterial contamination.

Transient transfection was performed according to the instructions of the Lipofectamine 3000 reagent (L3000008, Invitrogen, USA) [36]. Briefly, HCC cells were seeded on cell culture plates in triplicate. When grown to an appropriate confluence (30–50%), the cells were transfected with si-PART1.1, -PART1.2, -PART1.3, si-SLC7A11.1, -SLC7A11.2, -SLC7A11.3, siRNA negative control (siRNA-NC), miRNA mimics of miR-490-3p or miRNA mimics NC at a final concentration of 20 nM using the recommended volume of Lipofectamine 3000 reagent. Opti-MEM^™^ reduced serum medium was used for the first 6 h of transfection and then changed to DMEM complete medium. Cells were harvested after 24–72 h for subsequent assays. The sequences of the siRNAs and miRNA mimics are listed in Supplementary Table 1.

### Quantitative real-time PCR

Total RNA was extracted from cells using the TRIzol (EX1880, G-CLONE, China) method according to the standard TRIzol extraction procedure. For mRNAs and lncRNAs, 2 μg total RNA was reverse transcribed into cDNA by using the Servicebio First Strand cDNA Synthesis Kit (G3330, Servicebio, China). For miRNA analysis, reverse transcription was performed using the One Step miRNA cDNA Synthesis Kit (D1801, HaiGene, China) with random primers. RT-qPCR was performed using 2 × Fast SYBR Green qPCR Master Mix (A2250B, Servicebio, China) or RAPA3G SYBR Green qPCR Mix (A2250, HaiGene, China) on a LightCycler 96 real-time quantitative PCR instrument. GAPDH or U6 was used as internal control, and the 2^−ΔΔCt^ method was used for fold-change calculation. The primers used in this study are listed in Supplementary Table 1.

### Western blotting

Proteins from HCCLM3 and Huh7 cells transfected with siRNA, miRNA mimics, or negative controls were extracted using nuclear and cytosolic protein extraction kits (KGP150, Keygen, China) and quantified using a BCA kit (PA101, Biomed, China). Samples were subjected to sodium dodecyl sulfate-polyacrylamide gel electrophoresis **(**SDS-PAGE) and transferred to PVDF membranes (BS-PVDF, Biosharp, China). The PVDF membranes were then blocked with skimmed milk powder (5%) for 2 h at 25°C, before incubating with primary antibodies overnight at 4°C. The samples were further incubated at room temperature with secondary antibodies for 1.5 h and the protein bands were detected using an enhanced chemiluminescence solution (BL520A, Biosharp, China). The relative expression of proteins was analyzed using ImageJ software. Antibodies used in this study include anti-SLC7A11 (NB300, Novus, Chian), anti-N-cadherin (13116T, CST, USA), anti-vimentin (5741T, CST, USA), anti-E-cadherin (3195T, CST, USA), anti-Bcl2 (ab32124, Abcam, UK), anti-Caspase3 (ab32351, Abcam, UK), anti-Bax (ab32503, Abcam, UK), anti-NF-kB p65 (ab32536, Abcam, UK), anti-Erk1/2 (4370T, CST, USA), β-actin (BL005B, Biosharp, China).

### Malondialdehyde (MDA) assay

The concentration of MDA in cell lysates was determined using a lipid peroxidation assay kit (BC0025, Solarbio, China) according to the manufacturer’s instructions. Briefly, HCC cells transfected with siRNA-PART1 or siRNA NC were treated with 5 μM erastin for 24 h and lysed with an extraction solution. Subsequently, thiobarbituric acid (TBA) solution was added to the samples to generate the MDA-TBA adduct by reacting with MDA. The concentration of MDA in the samples was determined by measuring the absorbance of the MDA-TBA adduct at 520 nm.

### Iron assay

Huh7 and HCCLM3 cells transfected with siRNA-PART1 or siRNA NC were treated with 5 μM erastin for 24 h, and the level of intracellular ferrous iron (Fe^2+^) was determined using an iron assay kit according to the manufacturer’s instructions (BC4355, Solarbio, China).

### CCK-8 assay

HCCLM3 or Huh7 cells (5 × 10^3^ cells/well) were seeded in 96-well plates and cultured for 0, 24, 48, 72, and 96 h. At predetermined time points, 10 μl of CCK-8 reagent (GK10001-30, Glpbio, China) was added to the wells and incubated for an additional 2 h at 37°C. Finally, the absorbance at 450 nm was detected using a microplate reader (Molecular Devices, USA).

### Dual-luciferase reporter gene assay

The pmirGLO dual-luciferase miRNA target expression vector was used to quantitatively evaluate microRNA (miRNA) activity by inserting miRNA target sites 3′ of the firefly luciferase gene (luc2). Renilla luciferase (hRluc-neo) was used as the control reporter for normalization. The reporter vectors pmirGLO-PART1-WT and -MUT contain the predicted miRNA target sequences of the wild-type and mutant versions of PART1, respectively. The reporter vectors pmirGLO-SLC7A11-WT and -MUT contain the predicted wild-type and mutant versions of the 3′-UTR of SLC7A11 mRNA, respectively. For the dual luciferase assay, HCC cells were seeded at a density of 5 × 10^4^ cells/well in 24-well plates, and wild-type (-WT) or mutant (-MUT) luciferase reporter plasmids were transfected into the cells together with miR-490-3p mimics. After 48 h, the luciferase activity was measured using a luciferase reporter assay kit (KGAF040, Keygen, China).

### Cell apoptosis assay

After 72 h of transfection, 1 × 10^6^ of HCCLM3 and Huh7 cells were trypsinized and gently resuspended with 500 μl of binding buffer. Subsequently, 5 μl of propidium iodide (PI) and 5 μl of Annexin V-FITC (KGA108, Keygen, China) were added to the samples and incubated for 15 min in the dark. Apoptotic cells were detected using a FACS Calibur flow cytometer (BD Bioscience, USA), and the apoptosis ratio was analyzed using Flowjo v10 software.

### Cell cycle assay

HCC cells were harvested after transfection for 72 h and fixed in 70% (v/v) ethanol at 4°C for 4 h. After washing once with phosphate buffered saline (PBS), PI solution containing RNAase (DA0023, LEAGENE, China) was added and incubated for 30 min at 37°C, followed by detection by flow cytometry. The percentage of cells at different stages of the cell cycle was analyzed using ModFit 3.0.

### Transwell assay

Transwell assays were performed to evaluate the migration and invasion ability of HCC cells. Briefly, 4 × 10^4^ of HCC cells in 100 μl of medium were added to the upper chamber (precoated with 3 mg/ml of Matrigel for the invasion assay), and 600 μl of DMEM supplemented with 10% FBS was added to the lower culture wells. After incubation at 37°C for 48 h, the cells were fixed with 4% paraformaldehyde for 30 min and stained with 0.1% crystal violet for 2 h at room temperature. Non-migrated cells were gently removed with cotton swabs, and cells were counted in random fields (20×) using an inverted phase-contrast microscope.

### Wound healing assay

Approximately 2.5 × 10^5^ HCCLM3 and Huh7 cells were seeded in 24-well plates and incubated overnight to obtain a fully confluent monolayer of cells. Using a 200 μl pipette tip, the wells were slowly scratched and then the culture wells were washed three times with PBS. Subsequently, the cells were further cultured in 1 ml serum-free medium for 48 h. Wound healing was photographed under an inverted microscope at 0 and 48 h, and the percentage of wound healing was calculated using ImageJ software.

### Determination of sorafenib sensitivity in HCC

The sensitivity of HCC to sorafenib was determined using the CCK-8 assay (Glpbio, China, GK10001-30) according to the manufacturer’s instructions. Multiple concentrations of sorafenib were applied to HCC cells for 24 h to determine the IC50 values of HCCLM3 and Huh7. Then, siRNA-PART1 was transfected into the two HCC cell lines to silence PART1. The IC50 values were determined by the viability of the siRNA-PART1 and siRNA-NC cells under multiple concentrations of sorafenib intervention.

### Statistical analysis

Data are expressed as the mean ± standard deviation (SD). Unpaired two-tailed Student’s *t*-tests were used to compare the differences between the two groups. All statistical tests were performed using GraphPad Prism version 9.0 (GraphPad Software, Inc., USA). All experiments were repeated at least three times independently. *P* < 0.05 indicates statistically significant differences.

## RESULTS

### Prognostic ferroptosis-associated lncRNA-based ceRNA network in HCC

**Figure 1:**
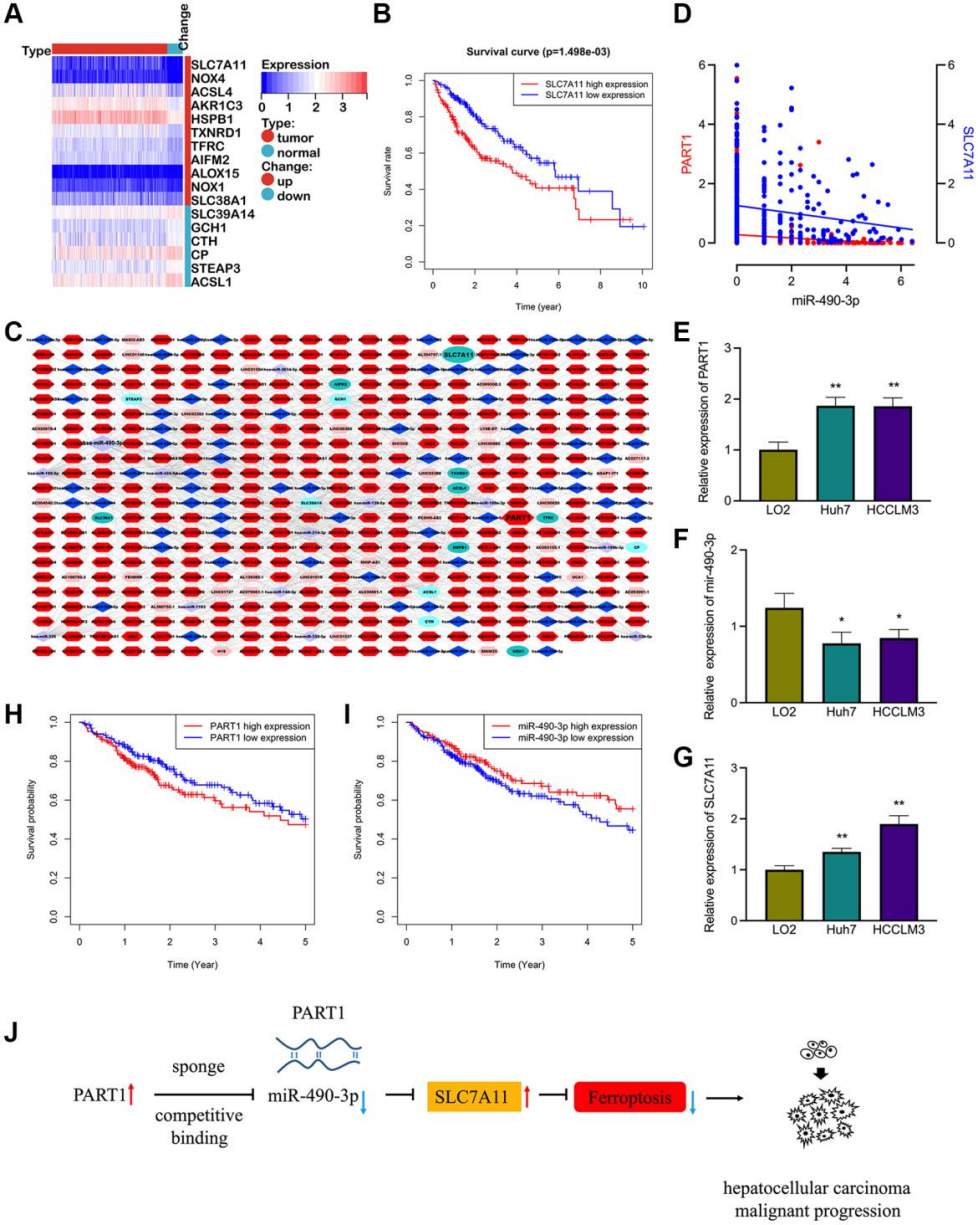
**The prognostic ferroptosis-associated lncRNA-based ceRNA network in HCC.** (**A**) Differentially expressed ferroptosis-associated genes in HCC. (**B**) Kaplan-Meier survival analysis for SCL7A11 in HCC patients. (**C**) The prognostic ferroptosis-associated ceRNA regulatory network based on lncRNA in HCC. Ellipse: LncRNA, diamond: miRNA, hexagon: mRNA, dark colors indicate up-regulated expressions and light colors indicate down-regulated expressions. (**D**) Correlation of miRNA-490-3p with the expression of PART1 and SLC7A11. (**E**–**G**) Relative expression of PART1, miRNA-490-3p, and SLC7A11 in HCC cell lines HCCLM and Huh7. (**H**–**I**) Kaplan-Meier survival analysis for PART1 and miRNA-490-3p in patients with HCC. (**J**) The PART1/miRNA/SLC7A11 axis in HCC derived from the ceRNA mechanism. ^*^p < 0.05, ^**^p < 0.01.

To screen for ferroptosis-related genes that are associated with the prognosis of patients with HCC, the ferroptosis molecular signature gene set (containing 64 ferroptosis-associated genes) was collected from the GSEA database. Based on the RNA sequencing data in TCGA database, 17 differentially expressed ferroptosis-related genes were identified in HCC, including 11 upregulated genes and six downregulated genes (Figure 1A). Of these, nine genes were associated with OS in patients with HCC by Kaplan–Meier survival analysis, namely SLC7A11, SALC38A1, HSPB1, CTH, TFRC, AIFM2, NOX4, ACSL4, and AKR1C3. With the exception of CTH, high expression of other genes was correlated with poor OS in patients with HCC (Figure 1B and Supplementary Figure 1). Furthermore, we screened 2,670 upregulated and 137 downregulated lncRNAs and 329 upregulated and 38 downregulated miRNAs between tumor and normal samples based on the gene expression data of patients with HCC in TCGA database. Subsequently, a prognostic ferroptosis-related lncRNA-based ceRNA network was established to explore the potential lncRNA/miRNA regulatory axis in HCC (Figure 1C). The ferroptosis-associated ceRNA network comprised eight prognosis-related mRNAs, 92 differentially expressed miRNAs, and 353 differentially expressed lncRNAs.

Among the numerous regulatory pathways, PART1 and SLC7A11 were identified as the predicted target genes of miR-490-3p. The expression of miR-490-3p was significantly reduced and negatively correlated with PART1 and SLC7A11 in HCC tissues (Figure 1D). The expression of PART1, miR-490-3p, and SLC7A11 was then validated by RT-qPCR in HCC cell lines. Compared to the normal hepatocyte line LO2, the expression of PART1 and SLC7A11 was significantly elevated, whereas miR-490-3p was significantly suppressed in the HCCLM3 and Huh7 HCC cell lines (Figure 1E–1G). Furthermore, low expression of miR-490-3p and high expression of PART1 were significantly associated with poorer prognosis in patients with HCC (Figure 1H, 1I). Therefore, we hypothesized that the PART1/miRNA-490-3p/ SLC7A11 axis represents a regulatory pathway involved in ferroptosis and that it may impact the prognosis of patients with HCC (Figure 1J). Our subsequent studies focused on validating the hypothesis through a series of *in vitro* experiments.

### PART1 promotes the proliferation of HCC cells and inhibits apoptosis

**Figure 2:**
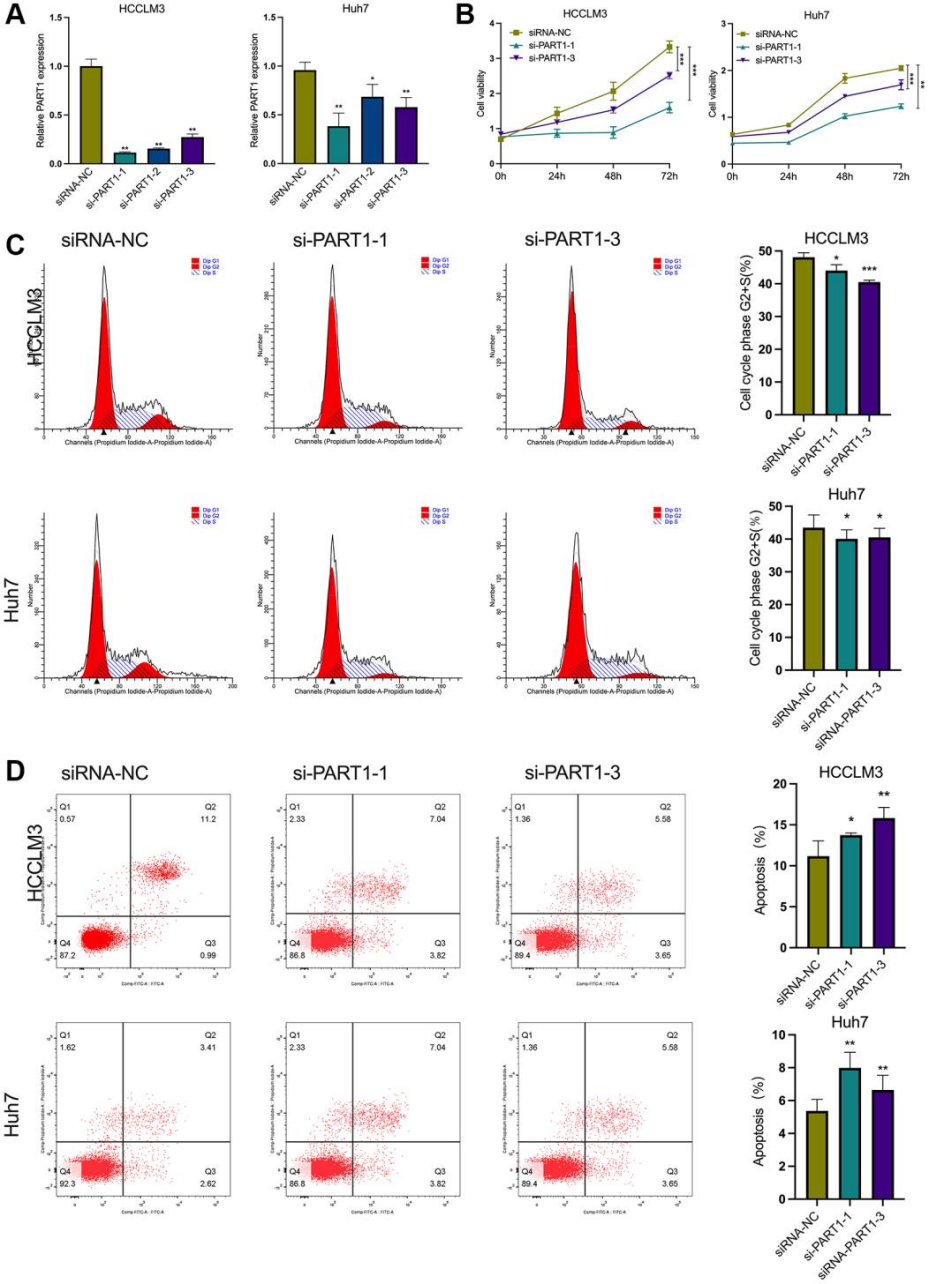
**PART1 promotes proliferation and inhibits apoptosis of HCC cells.** (**A**) The relative expression of PART1 was measured by RT-qPCR in HCCLM3 and Huh7 cells transfected with siRNA-PART1.1, -PART1.2, and -PART1.3. (**B**) CCK-8 assay was conducted to evaluate the proliferation of HCCLM3 and Huh7 cells transfected with siRNA-PART1.1 and -PART1.3. (**C**, **D**) The cell cycle and cell apoptosis rate of HCCLM3 and Huh7 cells transfected with siRNA-PART1.1 and -PART1.3 were assessed by flow cytometry analysis. ^*^p < 0.05, ^**^p < 0.01, ^***^p < 0.001.

**Figure 3:**
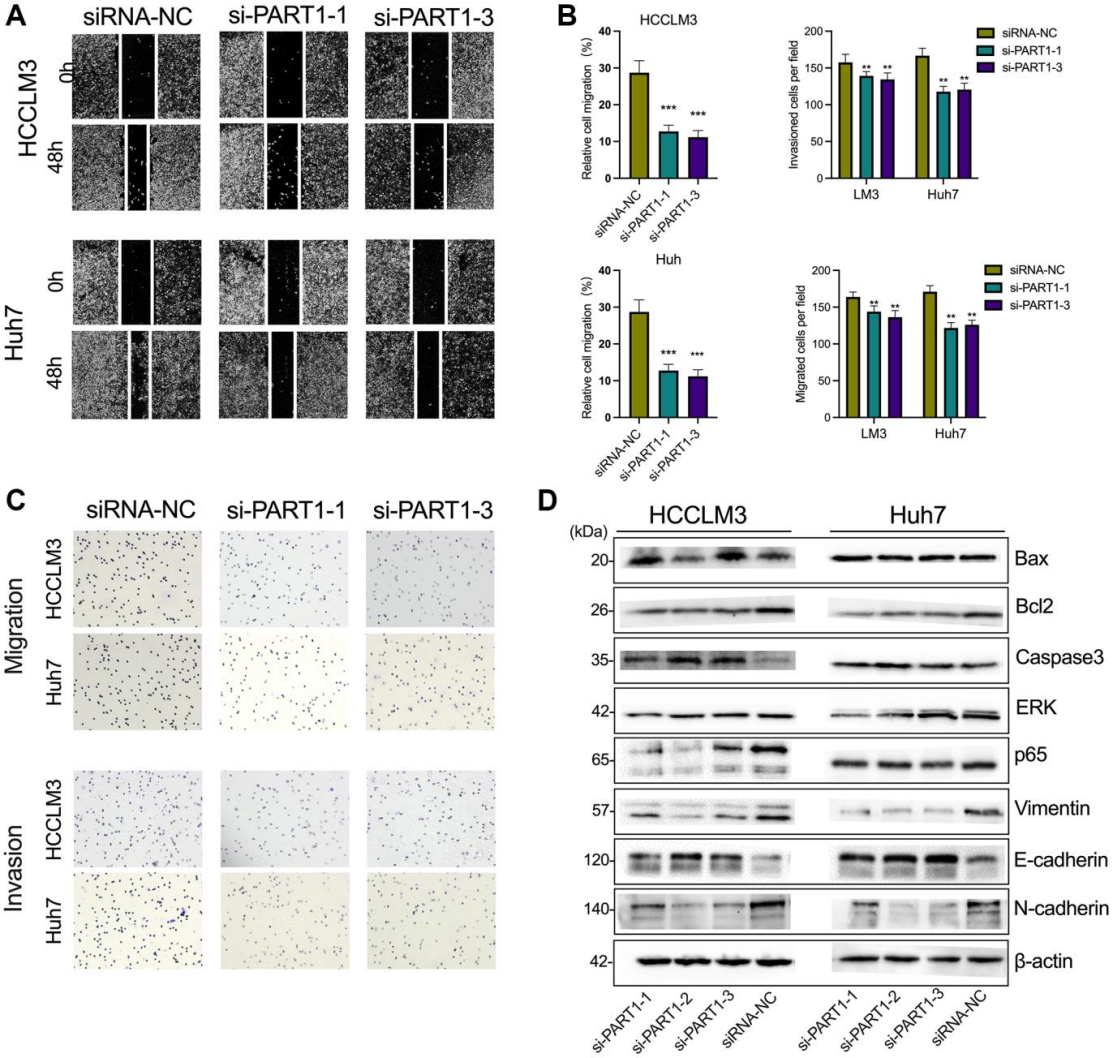
**Downregulation of PART1 inhibits migration, invasion, and metastasis of HCC cells.** (**A**) The migration and invasion were examined by wound healing assay in HCCLM3 and Huh7 cells transfected with siRNA-PART1.1 and -PART1.3. (**B**, **C**) The migration and invasion were illustrated by Transwell assay (40 × objective lens) in HCCLM3 and Huh7 cells transfected with siRNA-PART1.1 and -PART1.3. (**D**) The expression of apoptosis and EMT-related proteins (E-cadherin, vimentin, and N-cadherin) was detected by Western blotting. ^*^p < 0.05, ^**^p < 0.01, ^***^p < 0.001.

In this study, two human HCC cell lines, HCCLM3 and Huh7, were used for the following experiments. The HCCLM3 cell line has unique high metastatic properties and is widely used in basic and clinical studies on the pathogenesis of HCC as well as for antitumor drug screening. The Huh7 cell line is characterized by a fetoprotein (AFP)-positive, highly differentiated, epithelial-like adherent growth, which is suitable for studying carcinogenesis, metabolism, and regulation of gene expression. To evaluate the role of PART1 in the proliferation and apoptosis of HCC cells, lncRNA PART1 was knocked down by a single-gene small interfering RNA (siRNA) set, which comprised three pairs of candidate siRNAs. As shown in Figure 2A, si-PART1-1, -PART1-2, and -PART1-3 significantly decreased the expression of PART1 in the HCCLM3 and Huh7 HCC cell lines compared to siRNA-NC. Because of their higher knockdown efficiency, si-PART1.1 and -PART1-3 were selected for further experiments. Compared to the siRNA-NC group, the CCK-8 assay demonstrated that PART1 downregulation significantly inhibited the proliferation of Huh7 and HCCLM3 cells (Figure 2B), and flow cytometry revealed that PART1 knockdown reduced the proportion of cells in the G2/S phase (Figure 2C). In addition, we observed that the knockdown of PART1 led to an increase in apoptosis in HCCLM3 and Huh7 cells (Figure 2D). Furthermore, Western blotting showed that the expression of the anti-apoptotic factor Bcl2 was significantly downregulated in the si-PART1 group of HCC cells, whereas the expression of the pro-apoptotic factor Bax and cleaved caspase3 was significantly upregulated. Sustained activation of the NF-κB signaling pathway stimulates cell growth, inhibits apoptosis, leads to uncontrolled cell proliferation, and significantly promotes tumor metastasis. ERK signaling is closely associated with cell proliferation and can influence various tumor phenotypes. Western blotting showed that transfection of HCCLM3 or Huh7 cells with siRNA-PART1 significantly decreased the expression of p65 and ERK1/2 compared to the siRNA-NC group (Figure 3D). Taken together, these data suggest that PART1 promotes the proliferation of HCC cells and inhibits their apoptosis.

### Downregulation of PART1 inhibits the migration, invasion and metastasis of HCC cells

To elucidate the role of PART1 in HCC cell migration, invasion, and metastasis, wound healing, Transwells, and epithelial-mesenchymal transition (EMT)-related marker assays were performed. Wound healing assays showed a significant reduction in wound closure in HCCLM3 and Huh7 cells transfected with si-PART1 compared to the siRNA-NC group, indicating that PART1 knockdown significantly inhibited the migration of HCC cells (Figure 3A, 3B). Furthermore, the Transwell assay showed that the depletion of PART1 in HCCLM3 and Huh7 cells substantially hindered cell migration and invasion (Figure 3B, 3C). EMT activation is considered to be a critical process closely associated with cancer cell metastasis, during which epithelial cells acquire the characteristics of mesenchymal cells, with increased cell motility and migration capacity [37]. EMT is typically characterized by the upregulation of N-cadherin and Vimentin, followed by the downregulation of E-cadherin. As shown in Figure 3D, E-cadherin was significantly upregulated at the translational level in HCCLM and Huh7 cells transfected with si-PART1 compared to the siRNA-NC group, whereas N-cadherin and Vimentin were significantly downregulated. These results suggest that PART1 overexpression promotes the migration, invasion, and metastasis of HCC cells.

### PART1 protects HCC cells against erastin-induced ferroptosis

**Figure 4:**
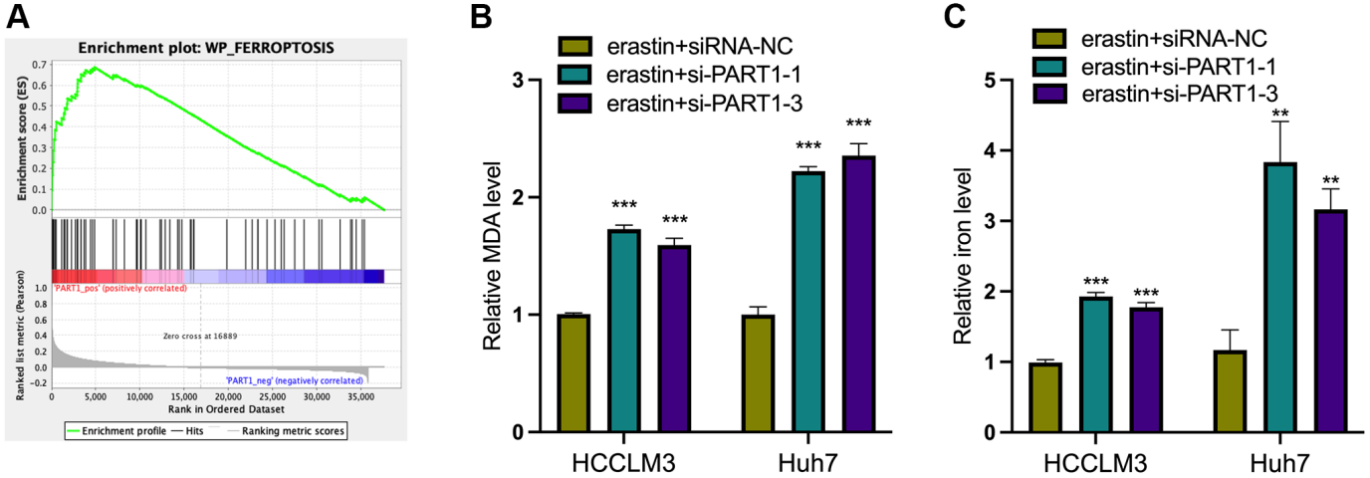
**PART1 protects HCC cells against erastin-induced ferroptosis.** (**A**) GSEA of the WP_Ferroptosis gene set was performed in HCC patients with different PART1 expression levels. (**B**) The relative levels of MDA in HCCLM3 and Huh7 cells transfected with siRNA-PART1.1 and -PART1.3 in the erastin-induced ferroptosis assay. (**C**) The relative levels of iron in HCCLM3 and Huh7 cells transfected with siRNA-PART1.1 and -PART1.3 in the erastin-induced ferroptosis assay. ^**^p < 0.01, ^***^p < 0.001.

Because ferroptosis is characterized by the accumulation of intracellular iron and lipid reactive oxygen species (ROS), to clarify the role of PART1 in ferroptosis in HCC cells, the lipid peroxidation marker MDA and intracellular iron were detected in erastin-induced ferroptosis. Erastin is a classical inducer of ferroptosis that blocks the entry of extracellular cysteine into the cell by inhibiting system xc^-^ activity, which in turn blocks the synthesis of intracellular GSH and weakens the antioxidant capacity of the cell, ultimately leading to the onset of ferroptosis. GSEA showed that PART1 expression was positively correlated with the enrichment of the WP_Ferroptosis gene set (Figure 4A). Compared to the siRNA-NC group, HCCLM3 or Huh7 cells transfected with si-PART1 showed significantly increases in the levels of MDA and intracellular iron (Figure 4B, 4C), suggesting that PART1 may be involved in regulating erastin-induced ferroptosis.

### Knockdown of SLC7A11 inhibited cell proliferation and facilitated ferroptosis in HCC cells

**Figure 5:**
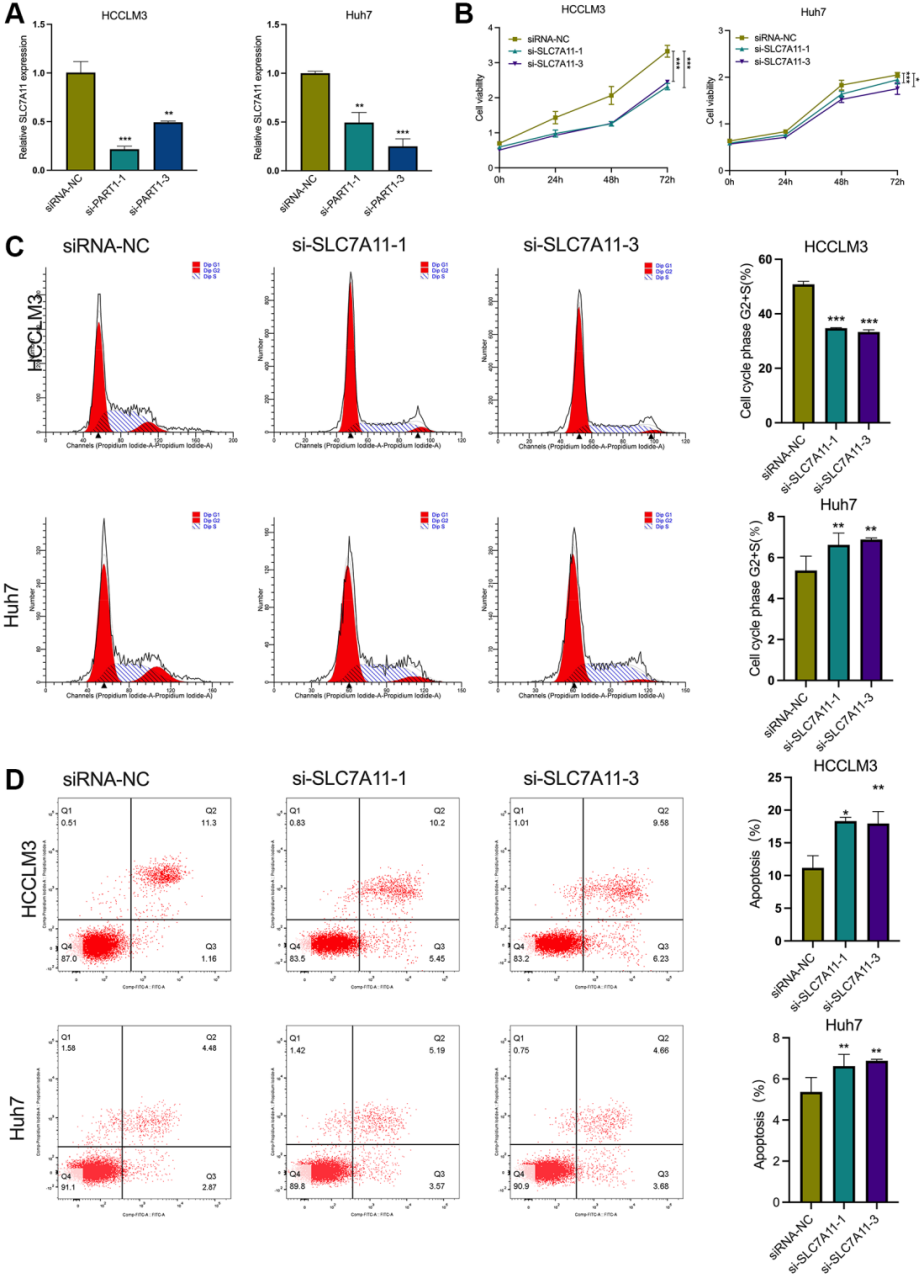
**SLC7A11 knockdown refrained cell proliferation and facilitated ferroptosis in HCC cells.** (**A**) The relative expression of SLC7A11 was measured by RT-qPCR in HCCLM3 and Huh7 cells transfected with siRNA-PART1.1 and -PART1.3. (**B**) CCK-8 assay was conducted to evaluate the proliferation of HCCLM3 and Huh7 cells transfected with siRNA-SLC7A11.1 and -SLC7A11.3. (**C**, **D**) The cell apoptosis and cell cycle were assessed by flow cytometry analysis in HCCLM3 and Huh7 cells transfected with siRNA-SLC7A11.1 and -SLC7A11.3. ^*^p < 0.05, ^**^p < 0.01, ^***^p < 0.001.

**Figure 6:**
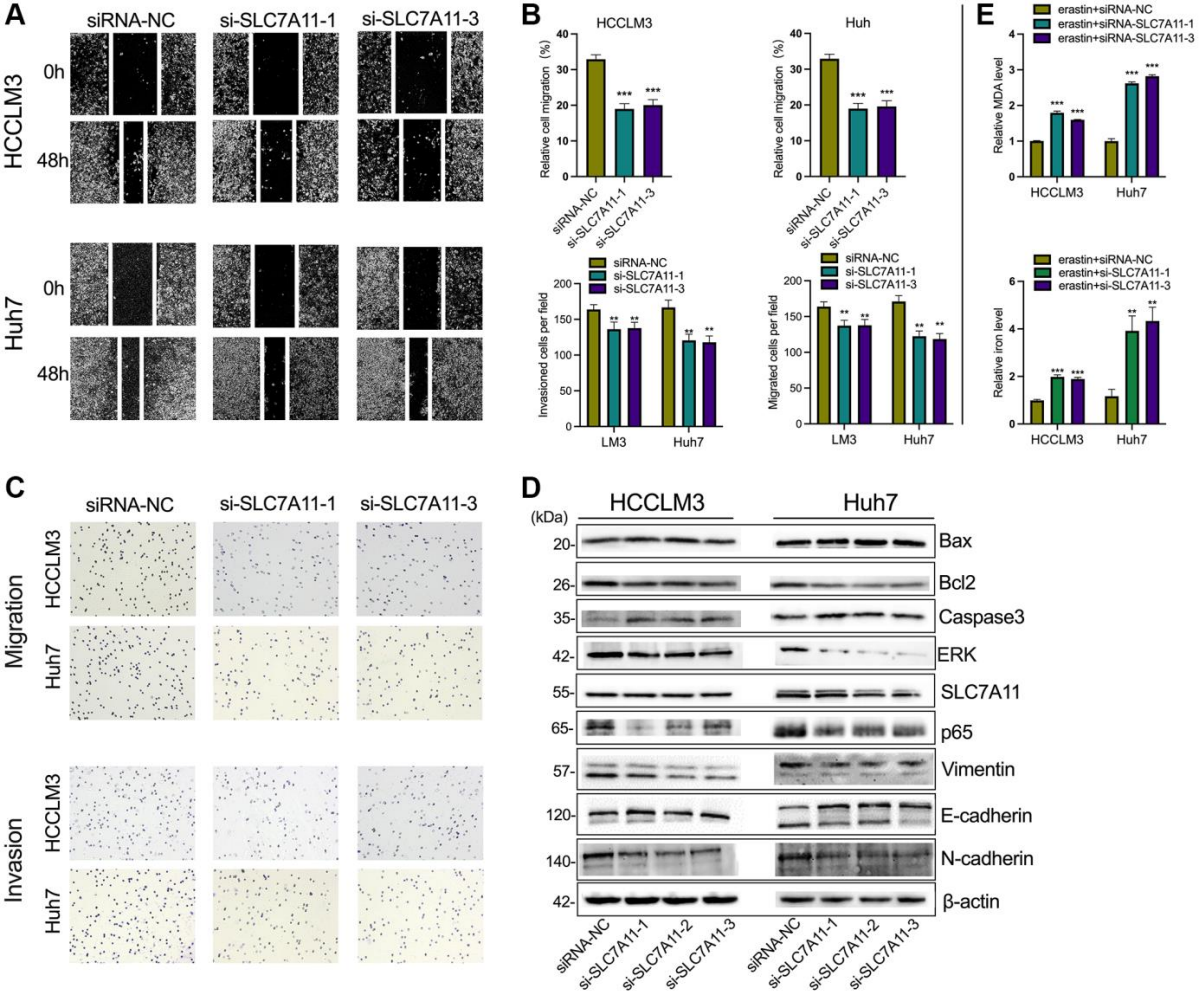
**The knockdown of SLC7A11 inhibited motility and promoted apoptosis in HCC cells.** (**A**–**C**) The migration and invasion were detected by Transwell assay (40 × objective lens) in HCCLM3 and Huh7 cells transfected with siRNA-SLC7A11.1 and -SLC7A11.3. The protein markers of the apoptosis were detected by Western blotting. (**D**) The expression of apoptosis and EMT-related proteins (E-cadherin, vimentin, and N-cadherin) was detected by Western blotting. (**E**) The relative levels of MDA and iron in HCCLM3 and Huh7 cells transfected with siRNA-SLC7A11.1 and -SLC7A11.3 in the erastin-induced ferroptosis assay. ^*^p < 0.05, ^**^p < 0.01, ^***^p < 0.001.

As mentioned above, PART1 may act as a ceRNA to regulate the expression of SLC7A11. To determine whether PART1 acts by regulating SLC7A11 expression, we first observed the effects of SLC7A11 knockdown on HCC cells. Compared to the negative control group, transient transfection of HCCLM3 and Huh7 cells with si-PART1 significantly reduced the expression of SLC7A11 at the transcript and translation levels (Figure 5A and Figure 6B). Transfection of HCCLM3 and Huh7 cells with si-SLC7A11 to reduce SLC7A11 expression significantly decreased cell viability, inhibited cell proliferation, attenuated migration and invasion, and increased apoptosis compared to the negative control group (Figure 5B–5D and Figure 6A–6C). Furthermore, impaired cell motility and increased apoptosis were verified by Western blotting. As shown in Figure 6D, the apoptosis-related proteins Bax and caspase3 were upregulated, whereas Bcl2, p65, and ERK1/2 were downregulated. Furthermore, E-cadherin was upregulated, whereas N-cadherin and Vimentin were downregulated in HCCLM3 and Huh7 cells transfected with si-SLC7A11 compared to the negative control group (Figure 6D). The levels of intracellular MDA and iron were significantly increased in HCCLM3 and Huh7 cells transiently transfected with si-SLC7A11 compared to the control group (Figure 6E). These results suggest that PART1 overexpression enhances the malignant phenotype of HCC and inhibits ferroptosis by increasing SLC7A11 expression.

### PART1 promotes HCC progression and inhibits ferroptosis by sponging miR-490-3p in HCC cells

**Figure 7:**
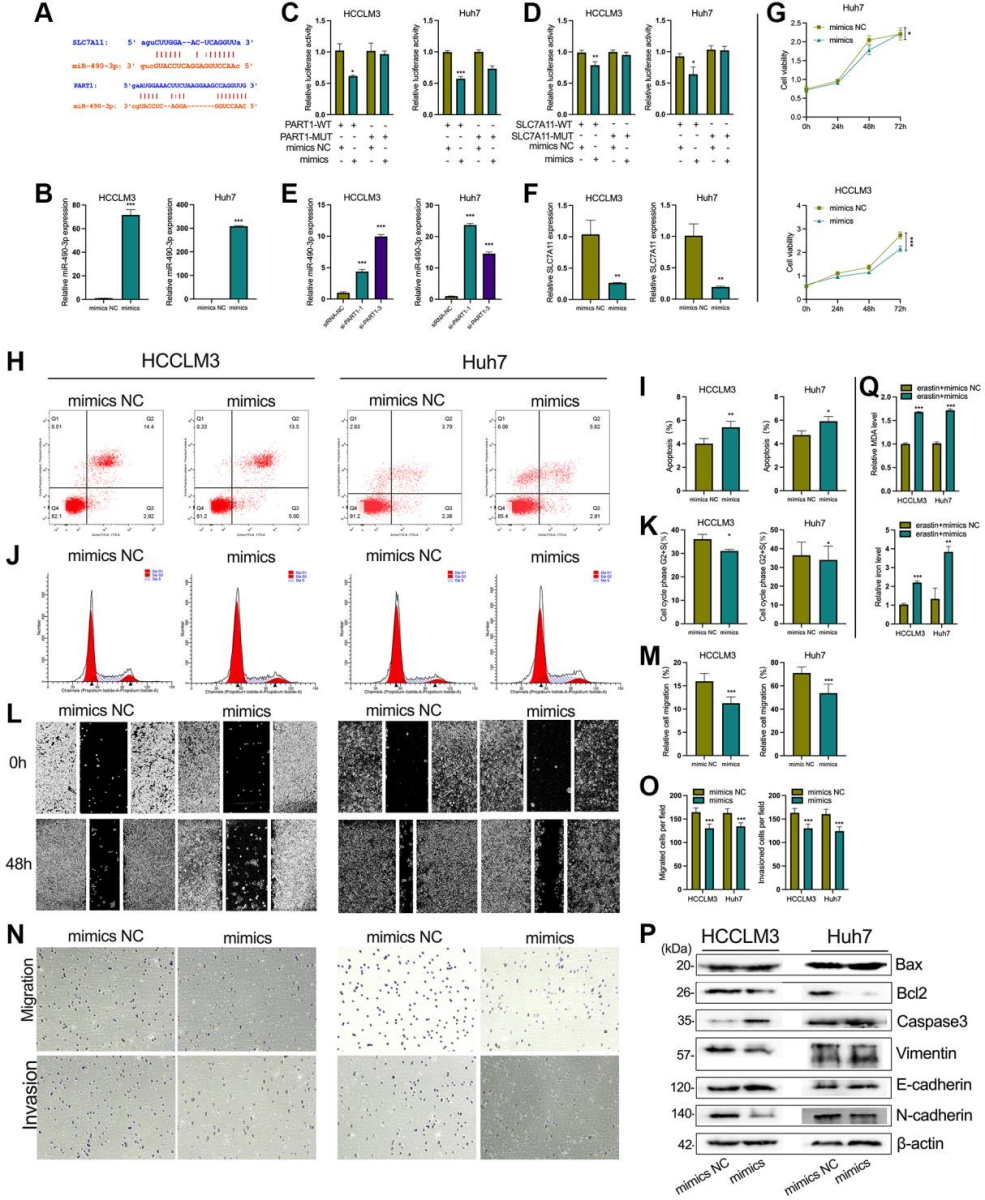
**PART1 promotes HCC progression and inhibits ferroptosis by sponging miR-490-3p.** (**A**) The interaction of miR-490-3p with PART1 and SLC7A11 predicted by ENCORI and LncBase v2 database. (**B**) The relative expression of miR-490-3p detected by RT-qPCR in HCCLM3 and Huh7 cells treated with miR-490-3p mimics or mimics NC. (**C**, **D**) Results of dual-luciferase reporter assay for miRNA-490-3p targeting PART1 and SLC7A11 mRNA 3'-UTR in HCCLM3 and Huh7 cells, respectively. (**E**) The relative expression of miR-490-3p in HCCLM3 and Huh7 cells transfected with siRNA-PART1.1 and -PART1.3. (**F**) The relative expression of SLC7A11 detected by qPCR in HCCLM3 and Huh7 cells treated with miR-490-3p mimics or mimics NC. (**G**) The cell proliferation was measured by the CCK-8 assays in HCCLM3 and Huh7 cells treated with miR-490-3p mimics or mimics NC. (**H**–**K**) The cell apoptosis and cell cycle were assessed by flow cytometry analysis in HCCLM3 and Huh7 cells treated with miR-490-3p mimics or mimics NC. (**L**, **M**) The migration was examined by wound healing assays in HCCLM3 and Huh7 cells treated with miR-490-3p mimics or mimics NC. (**N**, **O**) The cell migration and invasion were detected by Transwell assays (40 × objective lens) in HCCLM3 and Huh7 cells treated with miR-490-3p mimics or mimics NC. (**P**) The apoptosis and EMT-related proteins were detected by Western blotting. (**Q**) The relative levels of MDA and iron in HCCLM3 and Huh7 cells treated with miR-490-3p mimics or mimics NC in the erastin-induced ferroptosis assay. ^*^p < 0.05, ^**^p < 0.01, ^***^p < 0.001.

Mechanistically, bioinformatic predictions revealed that miR-490-3p can target both the PART1 and 3′-UTR of SLC7A11 mRNA (Figure 7A). As shown in Figure 7B, miR-490-3p expression was significantly upregulated when transfected with miR-490-3p mimics in HCCLM3 and Huh7 cells compared to the NC group. Next, dual luciferase reporter assays were performed to verify the predicted direct interactions between miR-490-3p and PART1 or SLC7A11. For the PART1/miR-490-3p interaction assay, miR-490-3p mimics (or mimics NC) and pmirGLO-PART1-WT (or -MUT) were co-transfected into HCCLM3 or Huh7. Compared to cells co-transfected with pmirGLO-PART1-WT and miR-490-3p mimics NC, the relative luciferase activity was significantly reduced in cells co-transfected with pmirGLO-PART1-WT and miR-490-3p mimics (Figure 7C). Similarly, the relative luciferase activity was significantly reduced in cells co-transfected with pmirGLO-SLC7A11-WT and miR-490-3p mimics compared to cells co-transfected with mimics NC (Figure 7D). These results indicate that miR-490-3p can directly interact with PART1 and SLC7A11. In addition, silencing PART1 in HCCLM3 and Huh7 cells significantly upregulated the expression of miR-490-3p while downregulating the expression of SLC7A11 (Figure 7E and Figure 5A). Meanwhile, miR-490-3p mimics significantly downregulated the expression of SLC7A11 (Figure 7F). The above results suggest that PART1 acts as an endogenous sponge for miR-490-3p in HCC cells to promote the expression of SLC7A11 and may promote HCC progression and inhibit ferroptosis through the miR-490-3p/SLC7A11 axis. The results of *in vitro* experiments showed that the upregulation of miR-490-3p expression in HCC cells by miR-490-3p mimics inhibited the cell proliferation of HCCLM3 and Huh7 cells (Figure 7G, 7J, 7K), induced apoptosis (Figure 7H, 7I), and enhanced the migration and invasive ability of HCC cells (Figure 7L–7O). Western blotting showed that the expression of Bax and caspase3 was significantly increased, while the anti-apoptotic factor Bcl2 was significantly decreased in the miR-490-3p mimics group compared to the mimics NC group (Figure 7P). Regarding the indicators of EMT, N-cadherin and Vimentin were significantly downregulated, while E-cadherin was upregulated in the miRNA-490-3p mimics group compared to the mimics NC group (Figure 7P). In addition, ROS and intracellular iron accumulated significantly in HCCLM3 and Huh7 cells transfected with miRNA-490-3p mimics (Figure 7Q).

### Targeting the PART1/miR-490-3p/SLC7A11 axis enhances the sensitivity of HCC cells to sorafenib

**Figure 8:**
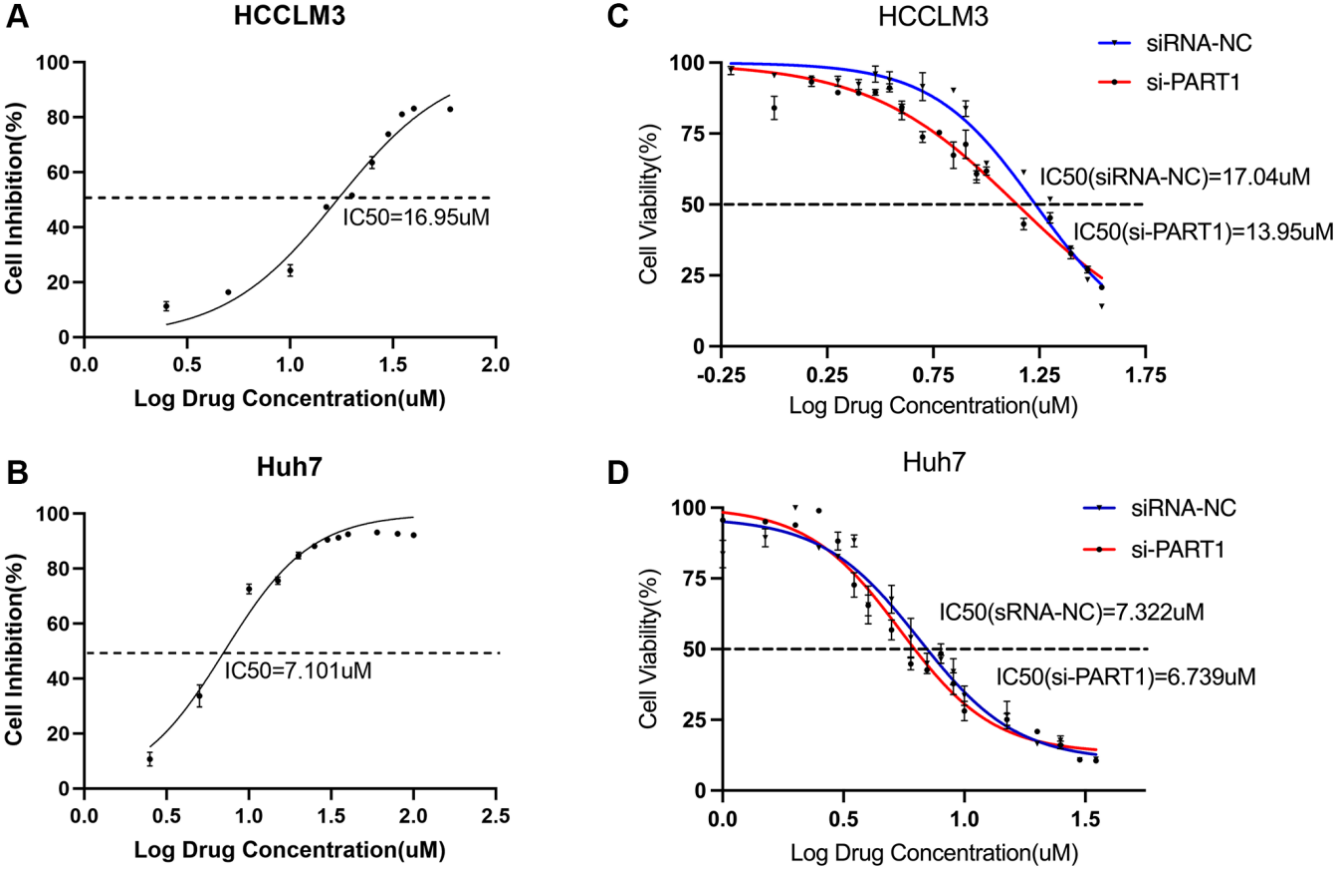
**Targeting the PART1/miR-490-3p/SLC7A11 axis enhances sorafenib sensitivity in HCC cells.** (**A**, **B**) The IC50 values were measured by the CCK-8 assays in the HCCLM3 and Huh7 cells. (**C**, **D**) CCK-8 assays tested the IC50 values of sorafenib in HCCLM3 and Huh7 cells transfected with siRNA-PART1.1.

Recent studies have shown that sorafenib is also a ferroptosis inducer that directly targets SLC7A11 and induces ferroptosis by blocking cystine uptake [38]. This toxic effect on HCC cells is partly dependent on the induction of ferroptosis; therefore, promoting sorafenib-induced ferroptosis may enhance the efficacy of sorafenib for treating HCC. In addition, ferroptosis plays an important role in the resistance of cancer cells to sorafenib. To investigate the role of PART1 in the sorafenib treatment of HCC, si-PART1 was transfected into Huh7 and HCCLM3 cells to silence PART1. Then, the effect of sorafenib on the cell viability of HCC cells was determined using the CCK-8 assay. Under normal conditions, the IC50 values of sorafenib against HCCLM3 and Huh7 were approximately 17 and 7 μM, respectively (Figure 8A, 8B). Compared to the siRNA NC group, transfection with si-PART1 increased the sensitivity of HCCLM3 and Huh7 to sorafenib. The IC50 values of sorafenib against HCCLM3 cells in the siRNA-NC group and si-PART1 group were 17.04 μM and 13.95 μM, respectively (Figure 8C). In Huh7 cells, the IC50 values of sorafenib was 7.322 μM and 6.739 μM in the siRNA NC group and si-PART1 groups, respectively (Figure 8D), suggesting that downregulation of PART1 expression increases the sensitivity of HCC cells to sorafenib.

## DISCUSSION

Increasing evidence suggests that lncRNAs are extensively involved in ferroptosis in various cancers and that they have a significant impact on the cancer progression and treatment sensitivity. LncRNA PART1 is aberrantly expressed in various human cancers and is associated with abnormal proliferation, migration, invasion, apoptosis and poor prognosis. As a ceRNA, PART1 can promote invasion, migration, and proliferation by targeting miR-4516 in breast cancer cells [39], regulate LRG1 expression in colorectal cancer by sponging miR-150-5p [40], enhance tumorigenesis by regulating the miR-503-5p/ FOXK1 axis in ovarian cancer [41], and promote proliferation while inhibiting apoptosis in bladder cancer, among others. In HCC, PART1 has been shown to promote HCC progression by targeting the miR-590-3p/HMGB2 [17], miR-149-5p/MAP2K1 [18], and miR-3529-3p/FOXC2 axis [16]. Here, we found that PART1 knockdown suppressed cell proliferation, invasion, migration, and metastasis while promoting apoptosis, which is consistent with the findings of previous reports. More importantly, to the best of our knowledge, this is the first study to uncover a novel mechanism by which PART1 suppresses ferroptosis and promotes HCC progression by regulating the miR-490-3p/SLC7A11 axis, where PART1 acts as a ceRNA of SLC7A11 sponging miR-490-3p to regulate the expression of SLC7A11, thus providing a deeper understanding of the ferroptosis mechanism in HCC.

Accumulating evidence confirms the critical role of ferroptosis in tumor progression and resistance to chemotherapy, radiotherapy, and even immunotherapy. Our study may have clinical significance for the diagnosis, prognostic assessment, and treatment of HCC. As reported in previous studies, PART1 is a promising biomarker for predicting the prognosis in pancreatic cancer [42], non-small cell lung cancer [43], and prostate cancer [44]. Additionally, miR-490-3p is known to function as a tumor suppressor in various cancers [45]. Indeed, reducing miR-490-3p expression is associated with poor prognosis of HCC [46], Helicobacter pylori-induced gastric cancer [47], non-small cell lung cancer [48], colorectal cancer [49], and triple negative breast cancer [50]. Furthermore, numerous studies have confirmed that SLC7A11 is overexpressed in a variety of cancers and is associated with poor patient prognosis [51]. Consistent with previous results, PART1, miR-490-3p and SLC7A1 were found to be aberrantly expressed in HCC and both were observed to be associated with poor prognosis in patients with HCC; thus, dysregulation of the PART1/miR-490-3p/SLC7A11 axis could serve as a diagnostic and prognostic marker for patients with HCC. Most patients with early-stage HCC have almost no symptoms and are often at the middle to late stage at the time of detection, thus screening for markers of early HCC is important for improving patient prognosis. It has been reported that miR-490-3p and SLC7A11 are promising molecular markers for early cancer diagnosis and prognosis [45, 52]. However, more clinical samples and data are required to determine the efficacy of miR-490-3p and SCL7A11 in diagnosing early HCC. Recently, Du et al. found that PART1 was secreted by exosomes in oral squamous cell carcinoma (OSCC). Overexpression of exosome-mediated lncRNA PART1 inhibited the viabilities, migration, and invasiveness of OSCC cells but facilitated OSCC cell apoptosis [53]. However, whether it can be secreted via exosomes in HCC cells and whether it affects patient prognosis remain unclear. Exosomes are an important source for obtaining serological marker; however, further studies are needed to determine whether PART1 can be secreted via exosomes and used as a serological marker in patients with HCC. In addition, as mentioned above, high expression of SLC7A11 has a significant effect on tumor chemotherapy, radiotherapy, and even immunotherapy. miR-490-3p functions as a tumor suppressor by suppressing the proliferation, apoptosis, autophagy, EMT, migration, and invasion of HCC [19, 20, 54, 55]. In this study, we identified SCL7A11 as a target gene of miR-490-3p. Overexpression of miR-490-3p significantly decreased the expression of SLC7A11 and increased ROS and iron accumulation, highlighting the potential of miR-490-3p to modulate the progression of HCC by regulating SLC7A11. It is worth exploring the possible combinations of treatments targeting miR-490-3p in the future for a better therapeutic outcome for patients with HCC.

This study has some limitation that warrant discussion. PART1 has more than one target gene, and other potential targets of PART1 should be investigated in future studies. For example, previous studies have shown that PART1 is associated with apoptosis in tumor cells. Yu et al. found that PART1 knockdown facilitated apoptosis of OSCC [56], and Zheng et al. found that PART1 downregulation triggered cell apoptosis in glioma cell lines via sponging miR-190a-3p and inactivating the PTEN/AKT pathway [57]. Consistent with these findings, we found that silencing PART1 decreased Bcl2 protein expression in HCC cells but increased Bax protein expression and promoted apoptosis in HCC cells. Bcl2 and Bax are important apoptosis-regulating genes. The homodimers of Bcl2 and Bax inhibit and induce apoptosis, respectively, whereas when they form a heterodimer, Bax can inhibit the anti-apoptotic function of Bcl2 and promote apoptosis. These data suggest that PART1 is an important lncRNA that mediates the cross-regulation between apoptosis and ferroptosis during the malignant progression of HCC. Current research suggests that PART1 plays a dual role in cancer by regulating cell proliferation, apoptosis, invasion and metastasis through multiple potential mechanisms [58]; that is, PART1 is upregulated in HCC, prostate cancer, and lung cancer and plays a role in promoting tumor growth. In contrast, PART1 is downregulated in esophageal squamous cell carcinoma and glioma and may suppress tumors. Thus, understanding the mechanisms that regulate PART1 expression in different tumors may be necessary for the development of novel therapeutic interventions against HCC and other tumors. In addition, the effects of PART1 and miR-490-3p on HCC growth and metastasis *in vivo*, especially on the sensitivity to sorafenib treatment, should be determined.

In conclusion, we found that PART1 promoted the malignant progression and alleviated ferroptosis in HCC through the miR-490-3p /SLC7A11 axis. Our findings provide new insights into the mechanism by which PART1 promotes HCC development of HCC and suggest that the PART1/miR-490-3p/SLC7A11 axis is a potential diagnostic biomarker and therapeutic target for HCC, which functions by modulating ferroptosis.

## Supplementary Materials

Supplementary Figure 1

Supplementary Table 1

## References

[1] Sung H, Ferlay J, Siegel RL, Laversanne M, Soerjomataram I, Jemal A, Bray F. Global Cancer Statistics 2020: GLOBOCAN Estimates of Incidence and Mortality Worldwide for 36 Cancers in 185 Countries. CA Cancer J Clin. 2021; 71:209–49. 10.3322/caac.21660 33538338

[2] Bebber CM, Müller F, Prieto Clemente L, Weber J, von Karstedt S. Ferroptosis in Cancer Cell Biology. Cancers (Basel). 2020; 12:164. 10.3390/cancers12010164 31936571 PMC7016816

[3] Conrad M, Pratt DA. The chemical basis of ferroptosis. Nat Chem Biol. 2019; 15:1137–47. 10.1038/s41589-019-0408-1 31740834

[4] Jiang N, Zhang X, Gu X, Li X, Shang L. Progress in understanding the role of lncRNA in programmed cell death. Cell Death Discov. 2021; 7:30. 10.1038/s41420-021-00407-1 33558499 PMC7870930

[5] Hassannia B, Vandenabeele P, Vanden Berghe T. Targeting Ferroptosis to Iron Out Cancer. Cancer Cell. 2019; 35:830–49. 10.1016/j.ccell.2019.04.002 31105042

[6] Li J, Cao F, Yin HL, Huang ZJ, Lin ZT, Mao N, Sun B, Wang G. Ferroptosis: past, present and future. Cell Death Dis. 2020; 11:88. 10.1038/s41419-020-2298-2 32015325 PMC6997353

[7] Liang C, Zhang X, Yang M, Dong X. Recent Progress in Ferroptosis Inducers for Cancer Therapy. Adv Mater. 2019; 31:e1904197. 10.1002/adma.201904197 31595562

[8] Batista PJ, Chang HY. Long noncoding RNAs: cellular address codes in development and disease. Cell. 2013; 152:1298–307. 10.1016/j.cell.2013.02.012 23498938 PMC3651923

[9] Karreth FA, Pandolfi PP. ceRNA cross-talk in cancer: when ce-bling rivalries go awry. Cancer Discov. 2013; 3:1113–21. 10.1158/2159-8290.CD-13-0202 24072616 PMC3801300

[10] Ulitsky I, Bartel DP. lincRNAs: genomics, evolution, and mechanisms. Cell. 2013; 154:26–46. 10.1016/j.cell.2013.06.020 23827673 PMC3924787

[11] Shi X, Sun M, Liu H, Yao Y, Song Y. Long non-coding RNAs: a new frontier in the study of human diseases. Cancer Lett. 2013; 339:159–66. 10.1016/j.canlet.2013.06.013 23791884

[12] Lee JT. Epigenetic regulation by long noncoding RNAs. Science. 2012; 338:1435–9. 10.1126/science.1231776 23239728

[13] Poliseno L, Marranci A, Pandolfi PP. Pseudogenes in Human Cancer. Front Med (Lausanne). 2015; 2:68. 10.3389/fmed.2015.00068 26442270 PMC4585173

[14] Tay Y, Rinn J, Pandolfi PP. The multilayered complexity of ceRNA crosstalk and competition. Nature. 2014; 505:344–52. 10.1038/nature12986 24429633 PMC4113481

[15] Chan JJ, Tay Y. Noncoding RNA:RNA Regulatory Networks in Cancer. Int J Mol Sci. 2018; 19:1310. 10.3390/ijms19051310 29702599 PMC5983611

[16] Weng Z, Peng J, Wu W, Zhang C, Zhao J, Gao H. Downregulation of PART1 Inhibits Proliferation and Differentiation of Hep3B Cells by Targeting hsa-miR-3529-3p/FOXC2 Axis. J Oncol. 2021; 2021:7792223. 10.1155/2021/7792223 34484336 PMC8410447

[17] Pu J, Tan C, Shao Z, Wu X, Zhang Y, Xu Z, Wang J, Tang Q, Wei H. Long Noncoding RNA PART1 Promotes Hepatocellular Carcinoma Progression via Targeting miR-590-3p/HMGB2 Axis. Onco Targets Ther. 2020; 13:9203–11. 10.2147/OTT.S259962 32982307 PMC7502387

[18] Zhou C, Wang P, Tu M, Huang Y, Xiong F, Wu Y. Long Non-Coding RNA PART1 Promotes Proliferation, Migration and Invasion of Hepatocellular Carcinoma Cells via miR-149-5p/MAP2K1 Axis. Cancer Manag Res. 2020; 12:3771–82. 10.2147/CMAR.S246311 32547213 PMC7248804

[19] Zhang H, Bao J, Zhao S, Huo Z, Li B. MicroRNA-490-3p suppresses hepatocellular carcinoma cell proliferation and migration by targeting the aurora kinase A gene (AURKA). Arch Med Sci. 2019; 16:395–406. 10.5114/aoms.2019.91351 32190151 PMC7069437

[20] Wang H, Yang G, Yu Y, Gu P. MicroRNA-490-3p suppresses the proliferation and invasion of hepatocellular carcinoma cells via targeting TMOD3. Oncol Lett. 2020; 20:95. 10.3892/ol.2020.11956 32831914 PMC7439154

[21] Wang H, Chen W, Jin M, Hou L, Chen X, Zhang R, Zhang J, Zhu J. CircSLC3A2 functions as an oncogenic factor in hepatocellular carcinoma by sponging miR-490-3p and regulating PPM1F expression. Mol Cancer. 2018; 17:165. 10.1186/s12943-018-0909-7 30470261 PMC6260990

[22] Chen W, Li K, Zhu K, Yan R, Cai QC, Li WH, Dang CX. RP11-81H3.2 Acts as an Oncogene via microRNA-490-3p Inhibition and Consequential Tankyrase 2 Up-Regulation in Hepatocellular Carcinoma. Dig Dis Sci. 2020; 65:2949–58. 10.1007/s10620-019-06007-5 31858324

[23] Koppula P, Zhuang L, Gan B. Cystine transporter SLC7A11/xCT in cancer: ferroptosis, nutrient dependency, and cancer therapy. Protein Cell. 2021; 12:599–620. 10.1007/s13238-020-00789-5 33000412 PMC8310547

[24] Liu T, Jiang L, Tavana O, Gu W. The Deubiquitylase OTUB1 Mediates Ferroptosis via Stabilization of SLC7A11. Cancer Res. 2019; 79:1913–24. 10.1158/0008-5472.CAN-18-3037 30709928 PMC6467774

[25] Sehm T, Rauh M, Wiendieck K, Buchfelder M, Eyüpoglu IY, Savaskan NE. Temozolomide toxicity operates in a xCT/SLC7a11 dependent manner and is fostered by ferroptosis. Oncotarget. 2016; 7:74630–47. 10.18632/oncotarget.11858 27612422 PMC5342691

[26] Hu Z, Mi Y, Qian H, Guo N, Yan A, Zhang Y, Gao X. A Potential Mechanism of Temozolomide Resistance in Glioma-Ferroptosis. Front Oncol. 2020; 10:897. 10.3389/fonc.2020.00897 32656078 PMC7324762

[27] Wang L, Leite de Oliveira R, Huijberts S, Bosdriesz E, Pencheva N, Brunen D, Bosma A, Song JY, Zevenhoven J, Los-de Vries GT, Horlings H, Nuijen B, Beijnen JH, et al. An Acquired Vulnerability of Drug-Resistant Melanoma with Therapeutic Potential. Cell. 2018; 173:1413–25.e14. 10.1016/j.cell.2018.04.012 29754815

[28] He J, Wang X, Chen K, Zhang M, Wang J. The amino acid transporter SLC7A11-mediated crosstalk implicated in cancer therapy and the tumor microenvironment. Biochem Pharmacol. 2022; 205:115241. 10.1016/j.bcp.2022.115241 36084707

[29] Drayton RM, Dudziec E, Peter S, Bertz S, Hartmann A, Bryant HE, Catto JW. Reduced expression of miRNA-27a modulates cisplatin resistance in bladder cancer by targeting the cystine/glutamate exchanger SLC7A11. Clin Cancer Res. 2014; 20:1990–2000. 10.1158/1078-0432.CCR-13-2805 24516043 PMC3974662

[30] Yadav P, Sharma P, Sundaram S, Venkatraman G, Bera AK, Karunagaran D. SLC7A11/ xCT is a target of miR-5096 and its restoration partially rescues miR-5096-mediated ferroptosis and anti-tumor effects in human breast cancer cells. Cancer Lett. 2021; 522:211–24. 10.1016/j.canlet.2021.09.033 34571083

[31] Sun C, Liu P, Pei L, Zhao M, Huang Y. Propofol Inhibits Proliferation and Augments the Anti-Tumor Effect of Doxorubicin and Paclitaxel Partly Through Promoting Ferroptosis in Triple-Negative Breast Cancer Cells. Front Oncol. 2022; 12:837974. 10.3389/fonc.2022.837974 35419287 PMC8996258

[32] Feng L, Zhao K, Sun L, Yin X, Zhang J, Liu C, Li B. SLC7A11 regulated by NRF2 modulates esophageal squamous cell carcinoma radiosensitivity by inhibiting ferroptosis. J Transl Med. 2021; 19:367. 10.1186/s12967-021-03042-7 34446045 PMC8393811

[33] Lei G, Zhang Y, Koppula P, Liu X, Zhang J, Lin SH, Ajani JA, Xiao Q, Liao Z, Wang H, Gan B. The role of ferroptosis in ionizing radiation-induced cell death and tumor suppression. Cell Res. 2020; 30:146–62. 10.1038/s41422-019-0263-3 31949285 PMC7015061

[34] Chen Q, Zheng W, Guan J, Liu H, Dan Y, Zhu L, Song Y, Zhou Y, Zhao X, Zhang Y, Bai Y, Pan Y, Zhang J, Shao C. SOCS2-enhanced ubiquitination of SLC7A11 promotes ferroptosis and radiosensitization in hepatocellular carcinoma. Cell Death Differ. 2023; 30:137–51. 10.1038/s41418-022-01051-7 35995846 PMC9883449

[35] Wang Z, Zhou C, Zhang Y, Tian X, Wang H, Wu J, Jiang S. From synergy to resistance: Navigating the complex relationship between sorafenib and ferroptosis in hepatocellular carcinoma. Biomed Pharmacother. 2024; 170:116074. 10.1016/j.biopha.2023.116074 38147732

[36] Müller V, Oliveira-Ferrer L, Steinbach B, Pantel K, Schwarzenbach H. Interplay of lncRNA H19/miR-675 and lncRNA NEAT1/miR-204 in breast cancer. Mol Oncol. 2019; 13:1137–49. 10.1002/1878-0261.12472 30803129 PMC6487715

[37] Barasch J. Genes and proteins involved in mesenchymal to epithelial transition. Curr Opin Nephrol Hypertens. 2001; 10:429–36. 10.1097/00041552-200105000-00021 11342809

[38] Lachaier E, Louandre C, Godin C, Saidak Z, Baert M, Diouf M, Chauffert B, Galmiche A. Sorafenib induces ferroptosis in human cancer cell lines originating from different solid tumors. Anticancer Res. 2014; 34:6417–22. 25368241

[39] Wang Z, Xu R. lncRNA PART1 Promotes Breast Cancer Cell Progression by Directly Targeting miR-4516. Cancer Manag Res. 2020; 12:7753–60. 10.2147/CMAR.S249296 32922076 PMC7457826

[40] Lou T, Ke K, Zhang L, Miao C, Liu Y. LncRNA PART1 facilitates the malignant progression of colorectal cancer via miR-150-5p/LRG1 axis. J Cell Biochem. 2020; 121:4271–81. 10.1002/jcb.29635 31898365

[41] Li B, Lou G, Zhang J, Cao N, Yu X. Repression of lncRNA PART1 attenuates ovarian cancer cell viability, migration and invasion through the miR-503-5p/FOXK1 axis. BMC Cancer. 2022; 22:124. 10.1186/s12885-021-09005-x 35100978 PMC8802513

[42] Hu X, Zhang L, Tian J, Ma J. Long non-coding RNA PART1 predicts a poor prognosis and promotes the malignant progression of pancreatic cancer by sponging miR-122. World J Surg Oncol. 2021; 19:122. 10.1186/s12957-021-02232-3 33865422 PMC8053290

[43] Zhu D, Yu Y, Wang W, Wu K, Liu D, Yang Y, Zhang C, Qi Y, Zhao S. Long noncoding RNA PART1 promotes progression of non-small cell lung cancer cells via JAK-STAT signaling pathway. Cancer Med. 2019; 8:6064–81. 10.1002/cam4.2494 31436388 PMC6792487

[44] Sun M, Geng D, Li S, Chen Z, Zhao W. LncRNA PART1 modulates toll-like receptor pathways to influence cell proliferation and apoptosis in prostate cancer cells. Biol Chem. 2018; 399:387–95. 10.1515/hsz-2017-0255 29261512

[45] Li Y, Tian D, Chen H, Cai Y, Chen S, Duan S. MicroRNA-490-3p and -490-5p in carcinogenesis: Separate or the same goal? Oncol Lett. 2021; 22:678. 10.3892/ol.2021.12939 34345303 PMC8323007

[46] Ding S, Jin Y, Hao Q, Kang Y, Ma R. LncRNA BCYRN1/ miR-490-3p/POU3F2, served as a ceRNA network, is connected with worse survival rate of hepatocellular carcinoma patients and promotes tumor cell growth and metastasis. Cancer Cell Int. 2020; 20:6. 10.1186/s12935-019-1081-x 31920461 PMC6945438

[47] Qu M, Li L, Zheng WC. Reduced miR-490-3p expression is associated with poor prognosis of Helicobacter pylori induced gastric cancer. Eur Rev Med Pharmacol Sci. 2017; 21:3384–8. 28829504

[48] Miao J, Gao Y, Guan W, Yu X, Wang Y, Jiang P, Yang L, Xu L, You W. High level of LncRNA MAPKAPK5-AS1 predicts poor prognosis and contributes to the malignant proliferation and EMT of non-small cell lung cancer via sponging miR-490-3p from HMGB2. Genes Genomics. 2023; 45:611–25. 10.1007/s13258-022-01339-5 36445573

[49] Wang B, Yin M, Cheng C, Jiang H, Jiang K, Shen Z, Ye Y, Wang S. Decreased expression of miR-490-3p in colorectal cancer predicts poor prognosis and promotes cell proliferation and invasion by targeting RAB14. Int J Oncol. 2018; 53:1247–56. 10.3892/ijo.2018.4444 29916545

[50] Fan H, Yuan J, Li X, Ma Y, Wang X, Xu B, Li X. LncRNA LINC00173 enhances triple-negative breast cancer progression by suppressing miR-490-3p expression. Biomed Pharmacother. 2020; 125:109987. 10.1016/j.biopha.2020.109987 32058222

[51] Lin W, Wang C, Liu G, Bi C, Wang X, Zhou Q, Jin H. SLC7A11/xCT in cancer: biological functions and therapeutic implications. Am J Cancer Res. 2020; 10:3106–26. 33163260 PMC7642655

[52] Liang Y, Su S, Lun Z, Zhong Z, Yu W, He G, Wang Q, Wang J, Huang S. Ferroptosis regulator SLC7A11 is a prognostic marker and correlated with PD-L1 and immune cell infiltration in liver hepatocellular carcinoma. Front Mol Biosci. 2022; 9:1012505. 10.3389/fmolb.2022.1012505 36267158 PMC9577028

[53] Du Y, Shuai Y, Wang H, Li H, Li Y. Exosome-mediated long noncoding RNA (lncRNA) PART1 suppresses malignant progression of oral squamous cell carcinoma via miR-17-5p/SOCS6 axis. Turk J Med Sci. 2023; 53:630–9. 10.55730/1300-0144.5625 37476905 PMC10388088

[54] Ou Y, He J, Liu Y. MiR-490-3p inhibits autophagy via targeting ATG7 in hepatocellular carcinoma. IUBMB Life. 2018; 70:468–78. 10.1002/iub.1715 29676845

[55] Zhang LY, Liu M, Li X, Tang H. miR-490-3p modulates cell growth and epithelial to mesenchymal transition of hepatocellular carcinoma cells by targeting endoplasmic reticulum-Golgi intermediate compartment protein 3 (ERGIC3). J Biol Chem. 2013; 288:4035–47. 10.1074/jbc.M112.410506 23212913 PMC3567655

[56] Yu Q, Du Y, Wang S, Zheng X. LncRNA PART1 promotes cell proliferation and inhibits apoptosis of oral squamous cell carcinoma by blocking EZH2 degradation. J Biochem. 2021; 169:721–30. 10.1093/jb/mvab026 33725092

[57] Jin Z, Piao L, Sun G, Lv C, Jing Y, Jin R. Long Non-Coding RNA PART1 Exerts Tumor Suppressive Functions in Glioma via Sponging miR-190a-3p and Inactivation of PTEN/AKT Pathway. Onco Targets Ther. 2020; 13:1073–86. 10.2147/OTT.S232848 32099409 PMC7007780

[58] Ran R, Gong CY, Wang ZQ, Zhou WM, Zhang SB, Shi YQ, Ma CW, Zhang HH. Long non-coding RNA PART1: dual role in cancer. Hum Cell. 2022; 35:1364–74. 10.1007/s13577-022-00752-y 35864416

