# Supplementary material for: PART1 facilitates tumorigenesis and inhibits ferroptosis by regulating the miR-490-3p/SLC7A11 axis in hepatocellular carcinoma
Source: Aging (Albany NY). 2024 Jul 5;16(14):11339–58. doi: 10.18632/aging.206009 (PMC11315397; doi:10.18632/aging.206009)
Supplement: Supplementary Figure 1 [file aging-16-206009-s001.pdf]

SUPPLEMENTARY FIGURE

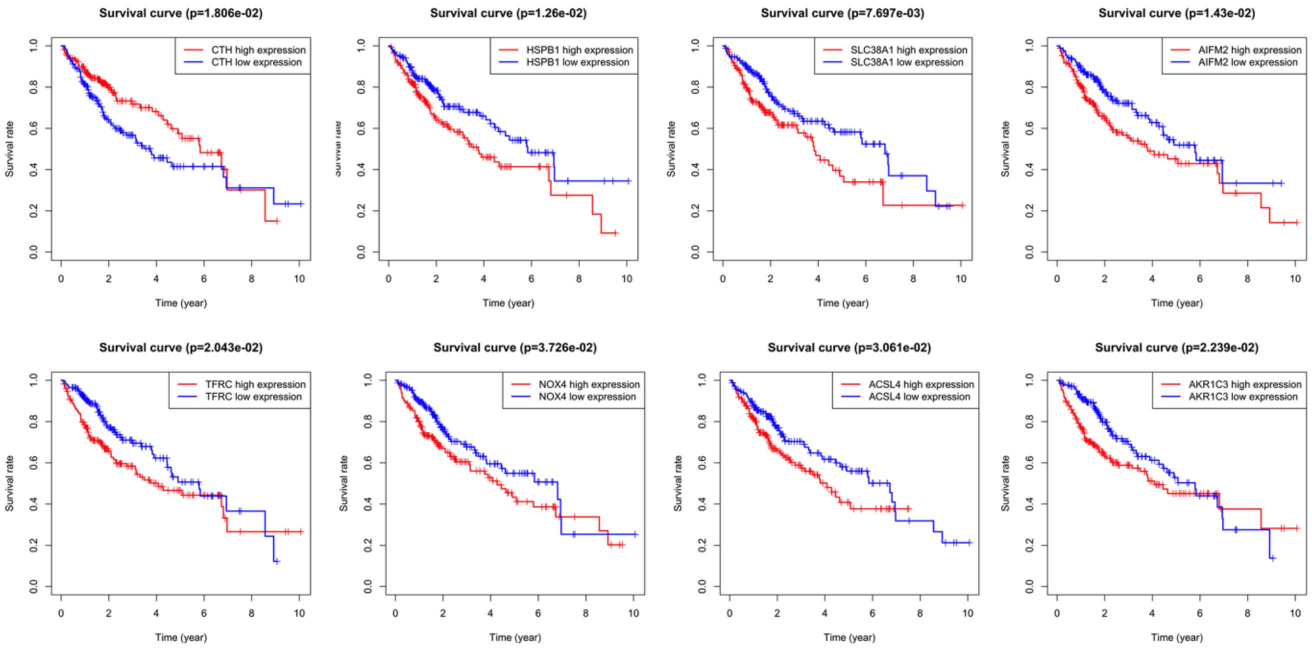

Supplementary Figure 1. Kaplan-Meier survival analysis for the differentially expressed prognosis-related ferroptosis-associated genes in patients with HCC.
