# Supplementary material for: PART1 facilitates tumorigenesis and inhibits ferroptosis by regulating the miR-490-3p/SLC7A11 axis in hepatocellular carcinoma
Source: Aging (Albany NY). 2024 Jul 5;16(14):11339–58. doi: 10.18632/aging.206009 (PMC11315397; doi:10.18632/aging.206009)
Supplement: Supplementary Table 1 [file aging-16-206009-s002.pdf]

SUPPLEMENTARY TABLE

Supplementary Table 1. The sequences of siRNAs and miRNA mimics.

Item	Sense (5′–3′) (including overhangs)	Antisense (5′–3′) (including overhangs)
siRNA-PART1-1	GGUGUGAAAUAAAGGUUAATT	UUAACCUUUAUUUCACACCTT
siRNA-PART1-2	GAAAGUUGUUGAAUAUAAATT	UUUAUAUUCAACAACUUUCTT
siRNA-PART1-3	GAGUUGACUUUGUGUUAUATT	UAUAACACAAAGUCAACUCTT
siRNA-SLC7A11-1	GGAAGAGAUUCAAGUAUUATT	UAAUACUUGAAUCUCUUCCTT
siRNA-SLC7A11-2	GGAGUUAUGUUUAAGUAAATT	UUUACUUAAACAUAACUCCTT
siRNA-SLC7A11-3	GAGUUAGUUUGGUAUUAAATT	UUUAAUACCAAACUAACUCTT
miR-490-3p mimics	CAACCUGGAGGACUCCAUGCUG	GCAUGGAGUCCUCCAGGUUGUU
**The sequences of primers**		
**Item**	**F**	**R**
PART1	CAGCCATCTCACCAGACACC	CAGCCTGAATCCTCAATAATCC
SLC7A11	TCTCCAAAGGAGGTTACCTGC	AGACTCCCCTCAGTAAAGTGAC
miR-490-3p	CAACCTGGAGGACTCCAT	TCCAGTTTTTTTTTTTTTTTCAGCA
